# Not Just Another Cause of Dyspnea: Common Complaint Leads to a Rare Diagnosis

**DOI:** 10.1155/2013/974085

**Published:** 2013-04-08

**Authors:** Hemant Goyal, Shitij Arora, Frantz Duffoo, Utpal Bhat, Rekha Bhandari, Sritha Rajupet, Kyaw Kyaw Lwin, Jigar Patel

**Affiliations:** ^1^Department of Internal Medicine, Mercer University School of Medicine, 707 Pine Street, Macon, GA 31201, USA; ^2^Division of Immunology, Elmezzi School of Molecular Medicine, North Shore-LIJ Health System, Manhasset, NY 11030, USA; ^3^Department of Internal Medicine, Kingsbrook Jewish Medical Center, Brooklyn, NY 11203, USA; ^4^Department of Internal Medicine, Wyckoff Heights Medical Center, Brooklyn, NY 11237, USA

## Abstract

A 62-year-old man with past medical history of type 2 diabetes mellitus (DM-2) and hypertension presented with progressive shortness of breath since three months. He was diagnosed with diabetic polyradiculopathy with diaphragmatic involvement and was started on intravenous immunoglobulin (IVIg) therapy. Rapid improvement was seen as evidenced by increased vital capacity and other pulmonary function parameters. Considering the patient's positive response to intravenous immunoglobulins (IVIg), this case strengthens the fact that diaphragmatic involvement in Type 2 Diabetes Mellitus can be a part of focal or polyneuropathy and that the pathogenesis is immune mediated.

## 1. Introduction

Amongst the neuromuscular diseases that result in respiratory failure, diabetic polyradiculopathy is a less known entity [1]. There is no specific treatment protocol for diabetic neuropathy, and the reported cases in the literature have been treated with either IVIg or “wait and watch” for spontaneous regression, which is the expected course of disease [2–4]. In some instances, topiramate has been reported as a method of treatment [5]. In the aforementioned cases, the patients had significant symmetric proximal muscle weakness and sensory changes of diabetic polyradiculopathy with pain, as described in diabetic amyotrophy. Here we describe a case of type 2 DM in which the patient presented with subacute respiratory failure and symmetric proximal and distal muscle weakness but with no sensory symptoms.

## 2. Case Report

A 62-year-old male with past medical history of type 2 DM (DM-2) and hypertension of 20 years presented to our emergency room with progressive dyspnea that had worsened gradually over the last three months. He was being treated in another facility with questionable diagnosis of congestive heart failure with incomplete response to diuretic therapy. There was no history of chest pain, cough, orthopnea, or paroxysmal nocturnal dyspnea. He was a nonsmoker and had used alcohol occasionally. There was no recent viral or flu-like illness. On careful questioning, he complained of difficulty in walking and weakness in his legs. The patient denied any back pain, fever, weight loss, bladder involvement, or pain and paresthesias in his extremities. Neurological examination revealed bilateral symmetric muscle weakness with power of 4/5 in upper proximal and distal extremities and 3/5 in lower proximal and distal extremities. There was no ptosis or gaze paresis. Cranial nerves I–XII were grossly intact. There was no evidence of atrophy of the hand muscles and fasciculations. Sensory exam revealed decreased pinprick sensation distal part of extremities. Deep tendon reflexes were graded one in both upper and lower extremities. His blood pressure on admission was 161/106 mm Hg. A fasting blood glucose done at emergency room triage was 229 mg/dL. The clinical and functional examinations did not correlate with the severity of dyspnea. Arterial blood gas (ABG) revealed respiratory insufficiency (pO_2_ of 60 mm Hg, pCO_2_ of 58 mm Hg, and SaO_2_ of 89%).

## 3. Hospital Course

Initially, chest radiograph was obtained which revealed normal lung fields with no evidence of fluid overload. Serum electrolytes and thyroid panel were found to be normal. *β*-natriuretic peptide level and 2-D echocardiogram results were inconsistent with heart failure. Adequate glycemic control was achieved with insulin, and serial ABGs remained unchanged on day 1. As the diagnosis leaned towards neuromuscular involvement, bilevel positive alveolar pressure (BiPAP) was started which was followed by serial vital capacities and negative inspiratory pressure (NIP). CSF analysis narrowed the differential to demyelinating neuropathy, which proved to be inconsistent with Gullian-Barré syndrome. EMG analysis revealed sensorimotor polyneuropathy that was predominantly axonal and demyelinating in nature (Table 1). In the meantime, the patient's vital capacity dropped to 0.7 liters with NIP of −26 cm H_2_O. Anti-paraneoplastic antibody screening with ANA and anti-MAG levels were noncontributory. On day 4, the CSF culture was negative. As diabetic polyradiculopathy was considered the most likely diagnosis, intravenous immunoglobulin IVIg 0.4 g/kg/day was started for five days. The patient tolerated the treatment well and showed both subjective and objective improvement as assessed by serial ABGs, vital capacity, and NIP assessment (Table 2). The patient was discharged to a rehabilitation center in stable respiratory condition with intermittent nasal oxygen insufflation and overnight BiPAP ventilation.

## 4. Discussion

Diabetic polyradiculopathy is not a commonly encountered cause of respiratory failure [6]. Amato and Barohn describe diabetic polyradiculopathy as two major subtypes, asymmetric painful and symmetric painless. The more commonly appreciated subtype amongst the two is asymmetric painful (also known as diabetic amyotrophy), which is more prevalent in type 1 DM patients [7]. The second major subtype, symmetric painless diabetic polyradiculopathy, presents over a period of weeks to months, and there is progressive painless weakness that evolves symmetrically in the proximal and distal muscles. This form of diabetic polyradiculopathy resembles idiopathic chronic inflammatory demyelinating polyneuropathy (CIDP) in its clinical features with increased CSF protein and electrophysiology [7]. However, further studies are needed to identify whether the presence of CIDP in a patient with symmetric painless diabetic polyradiculopathy is a coincidental finding or it represents a new distinct diabetic polyradiculopathy. The pathology of symmetric painless diabetic polyradiculopathy is controversial, and some authors attribute it to the coexistence of idiopathic CIDP. It has been found that symmetric painless diabetic polyradiculopathy is more common amongst type 1 diabetics, though it can be found in type 2 diabetics as it may have been the case with our patient [7]. Spontaneous regression has been suggested as the course for this disease with less inclination towards a possible autoimmune mechanism. However, studies have shown that cyclophosphamide, IVIg, plasma exchange, and azathioprine are beneficial in this form of neuropathy, suggesting an inclination towards autoimmune etiology [7–10].

Our case differs from the classical description of diabetic polyradiculopathy by two main features. First, respiratory failure as a presenting feature and, second, the rapid response to IVIg therapy both have not been documented in association with either subtype of diabetic polyradiculopathy. Our patient presented with subacute respiratory failure over months, and clinical improvement was seen as a rapid, almost dramatic response to IVIg treatment. This strongly suggests a positive immune-mediated response. We suggest the institution of immunomodulators early in the course of the disease, particularly if respiratory compromise is a component of the disease manifestation.

## Figures and Tables

**Table 1:** Sensory nerve conduction studies.

Nerve/Sites				Rec site			Response		
Left median (digit 2)				II			No		
Left ulnar (digit 5)				V			No		

EMG summary table									
	Spontaneous					MUAP			Recruitment
	Ia fibers	Fib	PSW	Fasc	H.F.	Amp	Dur	PPP	pattern

L gastroc.	N	None	None	None	None	N	N	N	Reduced
L tibialis anterior	N	None	None	None	None	N	N	N	Reduced
L flex carpi ulnaris	N	None	None	None	None	N	N	N	Reduced
L biceps	N	None	None	None	None	N	N	N	Reduced
L lumb PSP	N	None	None	None	None	N	N	N	N
R lumb PSP	N	None	None	None	None	N	N	N	N
L lumb PSP	N	None	None	None	None	N	N	N	N

Abbreviations: N: normal, Fib: fibrillations, PSW: polyspike wave, H.F.: high frequency, MUAP: motor unit action potential, Amp: amplitude, Dur: durations, PPP: polyphasic potential L: Left, R: Right, and Gastroc.: Gastrocnemius.

**Table 2:** Comparison of respiratory parameters before and after 5 doses of 0.4 g/kg/day IVIg therapy.

	Before IVIg	After IVIg
pO_2_	60 mm Hg	69 mm Hg
pCO_2_	58 mm Hg	48 mm Hg
sO_2_	90%	98%
Bicarbonate	36.3 mEq/L	32.6 mEq/L
Vital capacity	O.7 l	1.5 l
NIP	−25 cm H_2_O	−40 cm H_2_O
